# Regeneration of Tracheal Tissue in Partial Defects Using Porcine Small Intestinal Submucosa

**DOI:** 10.1155/2018/5102630

**Published:** 2018-02-26

**Authors:** Nelson Bergonse Neto, Lianna Ferrari Jorge, Julio C. Francisco, Bruna Olandoski Erbano, Barbara Evelin Gonçalves Barboza, Larissa Luvison Gomes da Silva, Marcia Olandoski, Katherine Athayde Teixeira de Carvalho, Luiz Felipe Pinho Moreira, Jose Rocha Faria Neto, Eltyeb Abdelwahid, Luiz Cesar Guarita-Souza

**Affiliations:** ^1^Experimental Laboratory of Institute of Biological and Health Sciences of Pontifical Catholic University of Paraná (PUCPR), Rua Imaculada Conceição 1155, 80215-901 Curitiba, PR, Brazil; ^2^Center of Cytopathology of Paraná (CITOPAR), Av. Sete de Setembro 5426, 80240-220 Curitiba, PR, Brazil; ^3^Cell Therapy and Biotechnology in Regenerative Medicine Research Group, Pelé Pequeno Príncipe Institute, Avenida Silva Jardim 1632, 80250-200 Curitiba, PR, Brazil; ^4^Heart Institute (InCor), University of São Paulo Medical School, LM, 11 São Paulo, SP, Brazil; ^5^Feinberg School of Medicine, Feinberg Cardiovascular Research Institute, Northwestern University, 303 E. Chicago Ave., Chicago, IL 60611, USA

## Abstract

**Background:**

Surgical correction of tracheal defects is a complex procedure when the gold standard treatment with primary end-to-end anastomosis is not possible. An alternative treatment may be the use of porcine small intestinal submucosa (SIS). It has been used as graft material for bioengineering applications and to promote tissue regeneration. The aim of this study was to evaluate whether SIS grafts improved tracheal tissue regeneration in a rabbit model of experimental tracheostomy.

**Methods:**

Sixteen rabbits were randomized into two groups. Animals in the control group underwent only surgical tracheostomy, while animals in the SIS group underwent surgical tracheostomy with an SIS graft covering the defect. We examined tissues at the site of tracheostomy 60 days after surgery using histological analysis with hematoxylin and eosin (H&E) staining and analyzed the perimeter and area of the defect with Image-Pro® PLUS 4.5 (Media Cybernetics).

**Results:**

The average perimeter and area of the defects were smaller by 15.3% (*p* = 0.034) and 21.8% (*p* = 0.151), respectively, in the SIS group than in the control group. Histological analysis revealed immature cartilage, pseudostratified ciliated epithelium, and connective tissue in 54.5% (*p* = 0.018) of the SIS group, while no cartilaginous regeneration was observed in the control group.

**Conclusions:**

Although tracheal SIS engraftment could not prevent stenosis in a rabbit model of tracheal injury, it produced some remarkable changes, efficiently facilitating neovascularization, reepithelialization, and neoformation of immature cartilage.

## 1. Introduction

The trachea is composed of highly specialized tissues, which confer rigid support, longitudinal coating, and a functional epithelial covering [1, 2]. As a result of this complex structure, the treatment of tracheal defects and effective regeneration following injury are difficult [3, 4].

Tracheal defects are caused by various acquired and congenital abnormalities, including trauma, tuberculosis, cancer, and idiopathic causes [5]. Corrective surgery for tracheal lesions remains a challenging procedure due to various potential complications, such as the formation of fistulae and tissue necrosis [6].

Treatment options for tracheal reconstruction depend on the defect size [7, 8]. It is known that the trachea, in cases of injury, can have its length reduced by half in adults and by about one third in young children [9]. The gold standard treatment is end-to-end primary anastomosis [10–12]. However, when defects exceed these limits, alternative treatments must be considered.

New technologies based on tissue engineering approaches have been proposed to assist regeneration of the trachea. Although diverse autologous and heterologous biomaterials have been used to repair tracheal lesions, most are neither effective nor reliable, especially for the long-term management of tracheal defects [13–17]. Porcine small intestinal submucosa (SIS) has been widely used in many areas of medicine as a graft material, including bladder and urethra reconstruction, heart valve replacement, diaphragmatic defect, and abdominal wall repair, amongst others, all of them with encouraging results [17–20]. Several studies have demonstrated SIS's ability to promote reepithelialization and complete infiltration of mesenchymal cells with new vascular growth [21–24].

Considering the difficulties encountered in the tracheal reconstruction process and the capacity of SIS to promote tissue regeneration, we carried out this study to evaluate whether SIS grafts could improve tissue regeneration of the trachea in a rabbit model of experimental tracheostomy.

## 2. Material and Methods

This is an experimental, interventional, and randomized study with 16 New Zealand white rabbits. Animals were randomly divided into two groups: the control group, which underwent only tracheostomy (*n* = 8), and the SIS group (*n* = 8), which underwent tracheostomy followed by SIS graft implantation at the tracheal defect site. The experiments were performed according to Law 6.638, May 8, 1979—Standards for the Practice, Teaching and Scientific Practice of Animal Vivisection. This project was presented to CEUA (Committee of Ethics in Research in Animal Use of the PUCPR) and approved under article number 640 (Annex 1).

### 2.1. Preparation of Porcine Small Intestinal Submucosa (SIS) Grafts

**Figure 1:**
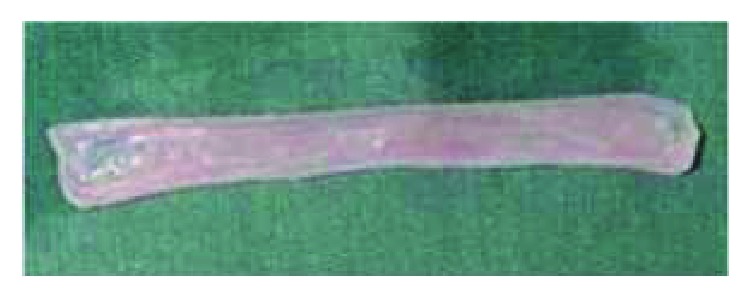
Mucosa of the inverted intestinal segment isolated.

The SIS was obtained from a slaughterhouse. A segment of jejunum 20 cm from the duodenal-jejunal flexure was removed from recently sacrificed, healthy pigs. Subsequently, the mesentery was removed from the jejunal segment. The intestinal segment was inverted by exposing the mucosa, which was removed by scraping with a bistoury. After reinversion of the tissue, the seromuscular layer was removed by the same procedure, leaving only the jejunal submucosa (Figure 1). The SIS was washed in 0.9% isotonic saline solution (JP/Equiplex) and stored in 10% neomycin sulfate solution. Decontamination was performed with 8% chlorine dioxide saline solution (Veromax 80®, Veros Chemical) at a dilution of 0.04% using a shaker (109M Bureau, New Ethics) for 24 hours [20]. The ultimate preparation was that of an acellular, decontaminated, porcine small intestinal submucosa composed of structural proteins, like collagen and elastin; glycoproteins (fibronectin and laminin); glycosaminoglycans and proteoglycans (hyaluronan, heparan sulfate, heparin, and dermatan sulfate); and matricellular proteins (thrombospondins, osteopontin, and tenascins).

### 2.2. Tracheostomy and Tracheal Reconstruction

The study included sixteen New Zealand male rabbits, with an average weight of 6 kg (±0.5 kg) and was conducted in the laboratory of experimental surgical technique of PUCPR.

**Figure 2:**
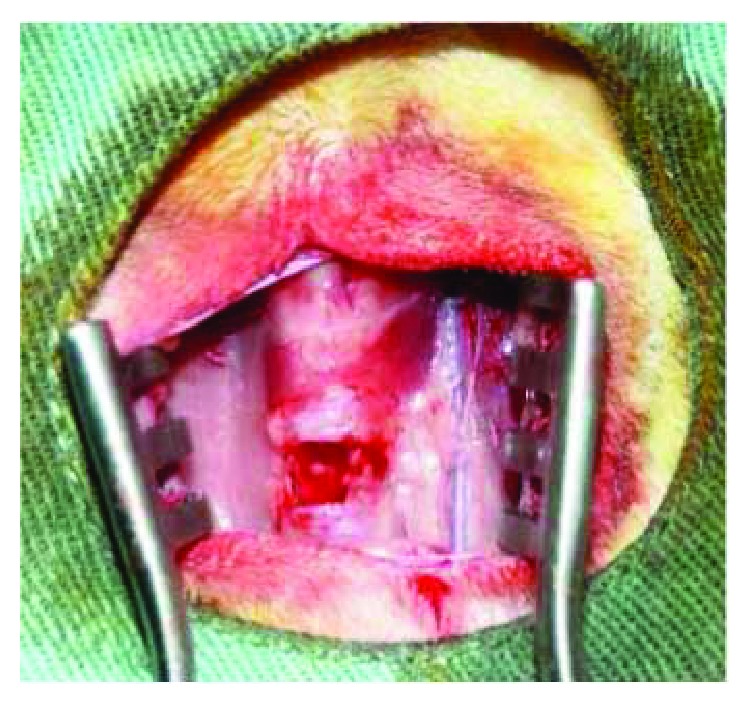
Trachea exposition with a 6 × 8 mm (48 mm^2^) defect made with a scalpel.

**Figure 3:**
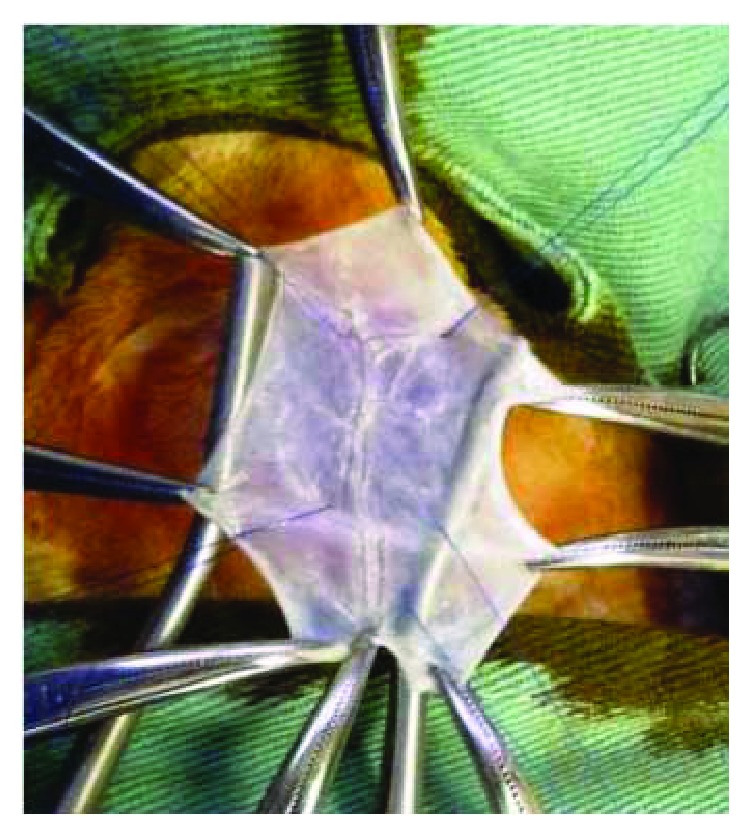
SIS group: implantation of the graft into the tracheal defect.

**Figure 4:**
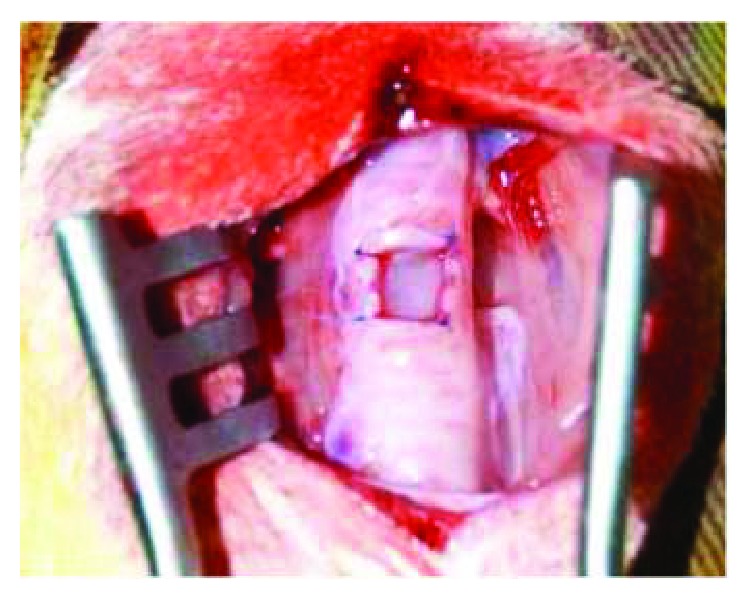
SIS group: defect covered by a rectangular graft supported by four points in the vertices with polypropylene 4.0.

The animals were anesthetized with xylazine (10 mg/kg), ketamine (20 mg/kg), acepromazine maleate (0.05 mg/kg), and propofol (5 mg/kg), and they were treated with the prophylactic antibiotic (gentamicin sulfate 5 mg/kg) intravenously. The necks of the rabbits were shaved and disinfected with 10% povidone-iodine and 70% ethanol in preparation for surgical tracheostomy. Local anesthesia was conducted with 1 mL of lidocaine hydrochloride (2%) immediately before the skin incision. A vertical incision was made at the neck, and the strap muscles were divided along the midline. After fully exposing the trachea, a 6 × 8 mm (48 mm^2^) tracheal defect was excised with a scalpel (Figure 2). In the SIS group, the defect was covered with a rectangular SIS graft supported at the four vertices and continuously sutured with polypropylene 4.0 (Figures 3 and 4). The strap muscles were replaced and reinforced over the graft, and the skin was sutured. In the animals of the control group, the tracheal defect was kept open and left to heal by secondary intention. The strap muscles were replaced and reinforced over the defect, and the skin was sutured.

Following surgery, rabbits were administered carprofen (2.2 mg/kg) for postoperative analgesia for three days.

Sixty days after undergoing surgical tracheostomy, rabbits were sedated and euthanized by intravenous administration of an overdose of pentobarbital (100 mg/kg).

**Figure 5:**
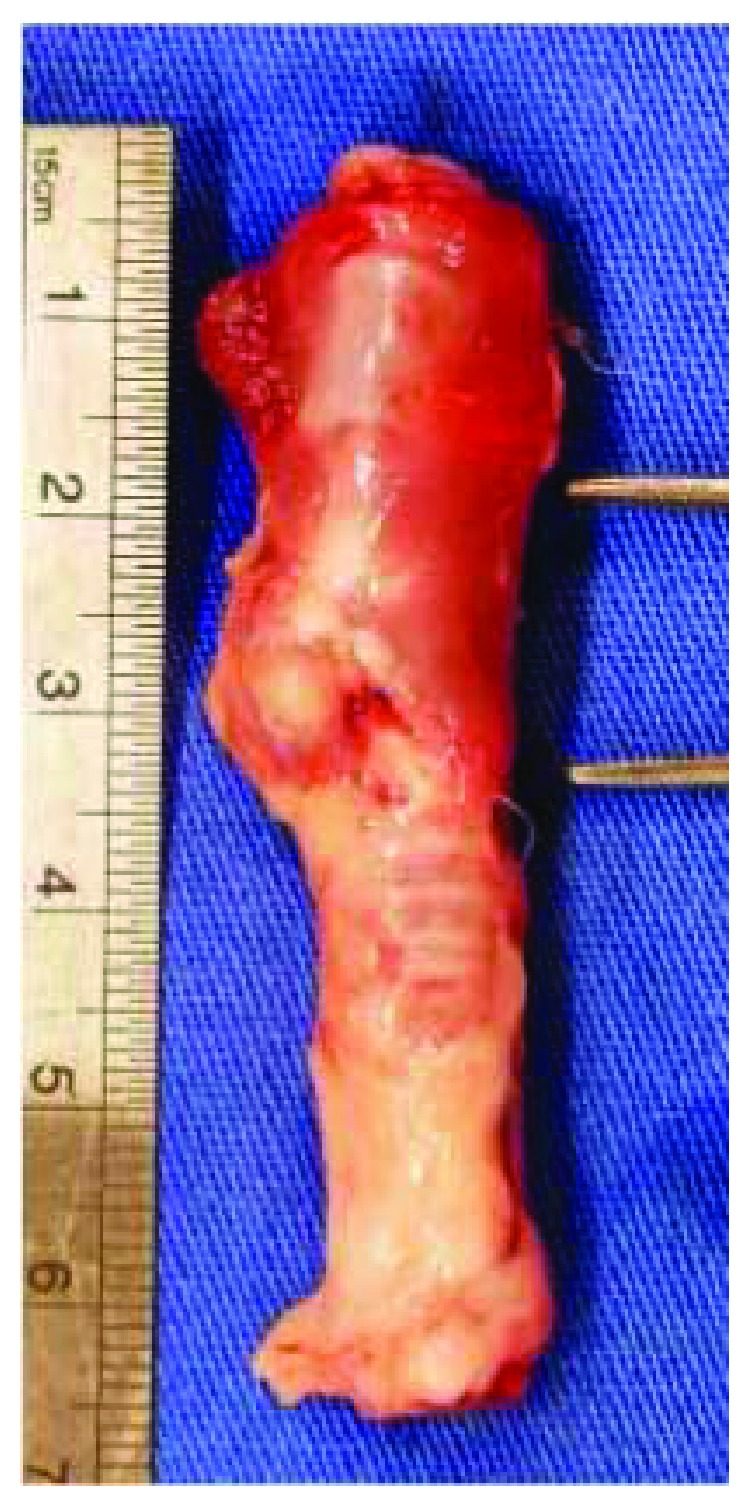
Trachea removed two centimeters above the main carina.

The trachea of each animal was dissected from two centimeters above the main carina and removed for histopathological analysis (Figure 5). The tracheas were fixed with 10% formaldehyde for 72 hours. Dehydration of the samples was performed by successive baths of alcohol (concentrations of 70%, 80%, and 90%) and three baths of 100% ethanol for 1 hour. Following dehydration, the samples were embedded in liquid paraffin using two baths at 65°C in the same equipment. After cooling, histological sections were taken by means of a microtome (Leica model RM 2145, Solms, Germany).

### 2.3. Analysis of Perimeter and Area of the Tracheal Defects

**Figure 6:**
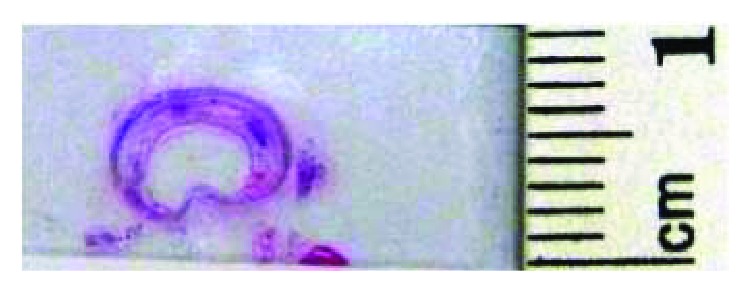
Photograph of the macroscopic section of the tracheal defect to analyze the morphology of the tracheal lumen.

Tracheal sections were macroscopically photographed to analyze the morphology of the tracheal lumen after surgery. The perimeters and areas of tracheal lumens in the defect regions of all sections were measured using Image-Pro PLUS 4.5 (Media Cybernetics) (Figure 6). In order to avoid differential operator bias, the same individual measured the variables three times, and the average values were taken for analysis. All photographs were taken with the same parameters, and a numerical scale was added to evaluate the defect dimensions in millimeters and square millimeters.

The results of the measurements of circumference and area of the defects were calculated as mean, median, minimum, maximum, and standard deviation values. Student's *t*-test for independent samples was used to compare between groups. Qualitative variables were compared using the Fisher exact test. *p* values < 0.05 were considered statistically significant. Data were analyzed with IBM SPSS Statistics V20 software.

### 2.4. Histological Analysis

Histological sections were stained with hematoxylin and eosin (H&E) and examined by light microscopy for identification of inflammatory reaction, fibrosis, neovascularization, and the presence of tissue regeneration characterized by evidence of reepithelialization and formation of new islands of cartilage.

Tissue regeneration was classified as present or absent. Other variables were also classified as present or absent. However, the present groups have also been divided into discreet, moderate, or severe presence for future analysis. The Fisher exact test was used to compare qualitative variables between the experimental groups. *p* values < 0.05 were considered statistically significant.

## 3. Results

### 3.1. Defect Perimeter and Area

**Figure 7:**
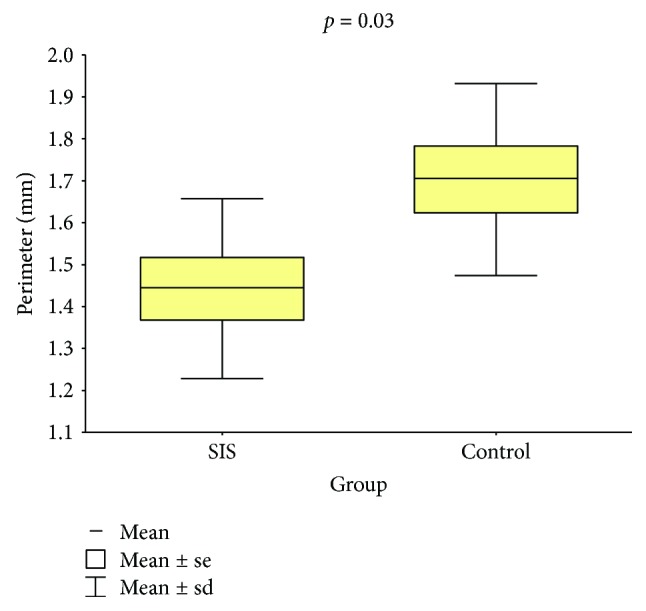
Tracheal defect perimeters of the groups.

**Figure 8:**
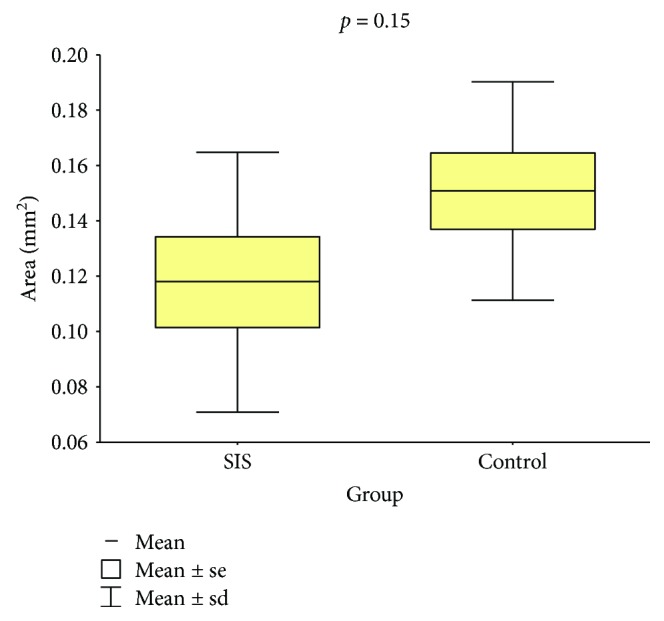
Tracheal defect areas of the groups.

Following a recovery period of 60 days, the average perimeter and area of the tracheal defects in control and SIS group animals were measured and compared (Tables 1 and 2; Figures 7 and 8). The average perimeter of the tracheal defect in the SIS group was 15.3% smaller than that of the control group, and the difference was statistically significant (*p* = 0.035). Similarly, the average area of the tracheal defect in the SIS group was 21.8% smaller than that in the control group, but this decrease was not statistically significant (*p* = 0.151).

### 3.2. Histological Analysis

The presence of inflammatory tissue, fibrosis, neovascularization, and tissue regeneration in the tracheal defect of control and SIS group animals was compared.

**Figure 9:**
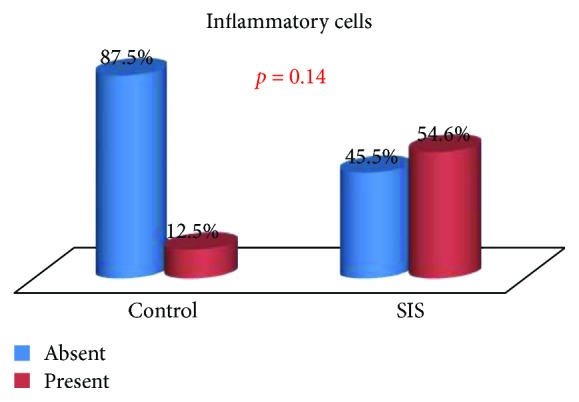
Inflammatory cells: group comparison.

Although inflammatory cells were observed at the site of the graft in 54.5% of the SIS group animals as against 12.5% of the control group animals, the difference was not statistically significant (*p* = 0.147) (Figure 9). Necrosis was not observed.

**Figure 10:**
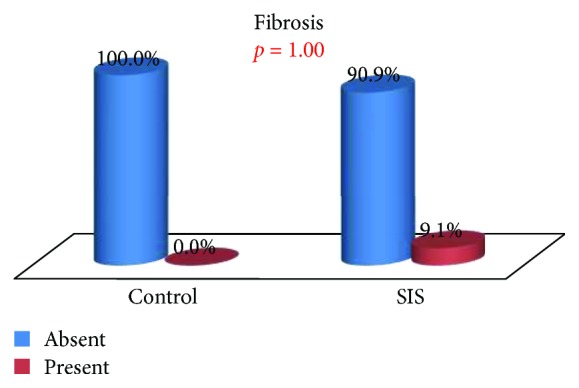
Fibrosis: group comparison.

**Figure 11:**
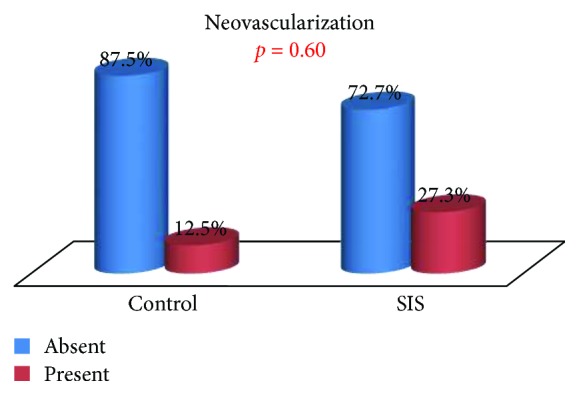
Neovascularization: group comparison.

There were also no statistically significant differences in fibrosis or neovascularization, although they were preferentially observed in the SIS group [9.09% against 0.0% (*p* = 1.000) and 27.27% against 12.5% (*p* = 0.603), resp.] (Figures 10 and 11). The SIS group presented evidence for the growth of new vessels in 3 of 8 animals.

**Figure 12:**
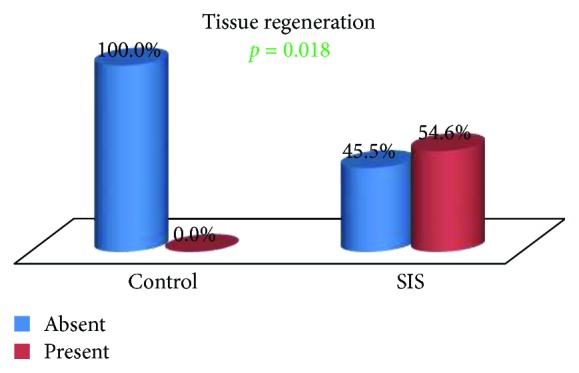
Tissue regeneration: group comparison.

**Figure 13:**
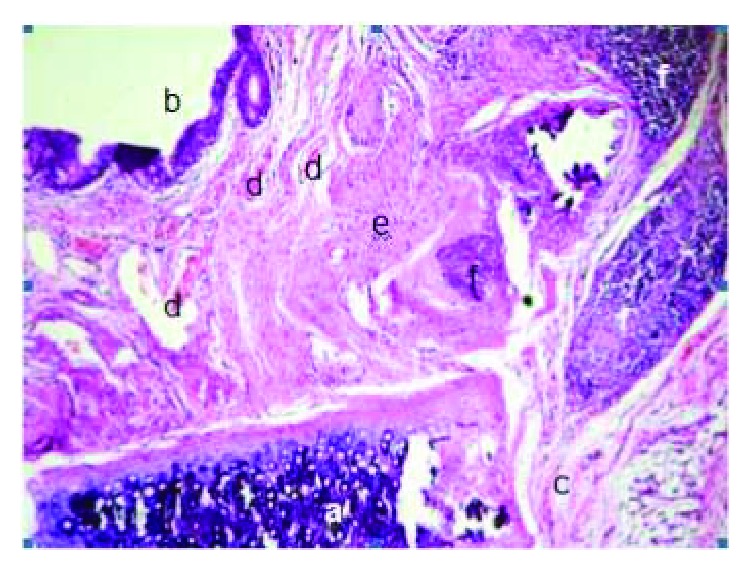
Control group without cartilaginous tissue regeneration (newly formed cartilage) at the site of the tracheal defect, H&E (100x): (a) mature cartilaginous tissue, (b) pseudostratified ciliated epithelium, (c) connective tissue, (d) blood vessels, (e) fibrosis, and (f) acute and chronic inflammatory tissue.

**Figure 14 fig14:**
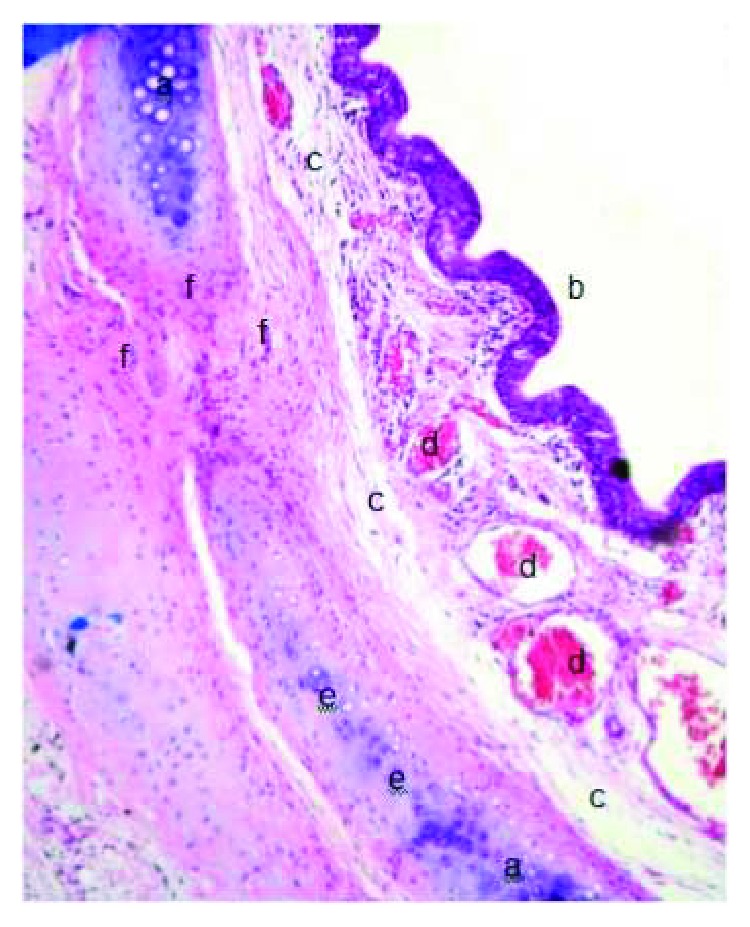
Tissue regeneration in the SIS group with reepithelialization, neovascularization, and newly formed cartilage, H&E (40x): (a) mature cartilaginous tissue, (b) pseudostratified ciliated epithelium, (c) connective tissue, (d) blood vessels, (e) immature cartilaginous tissue (newly formed cartilage), and (f) fibrosis.

**Figure 15 fig15:**
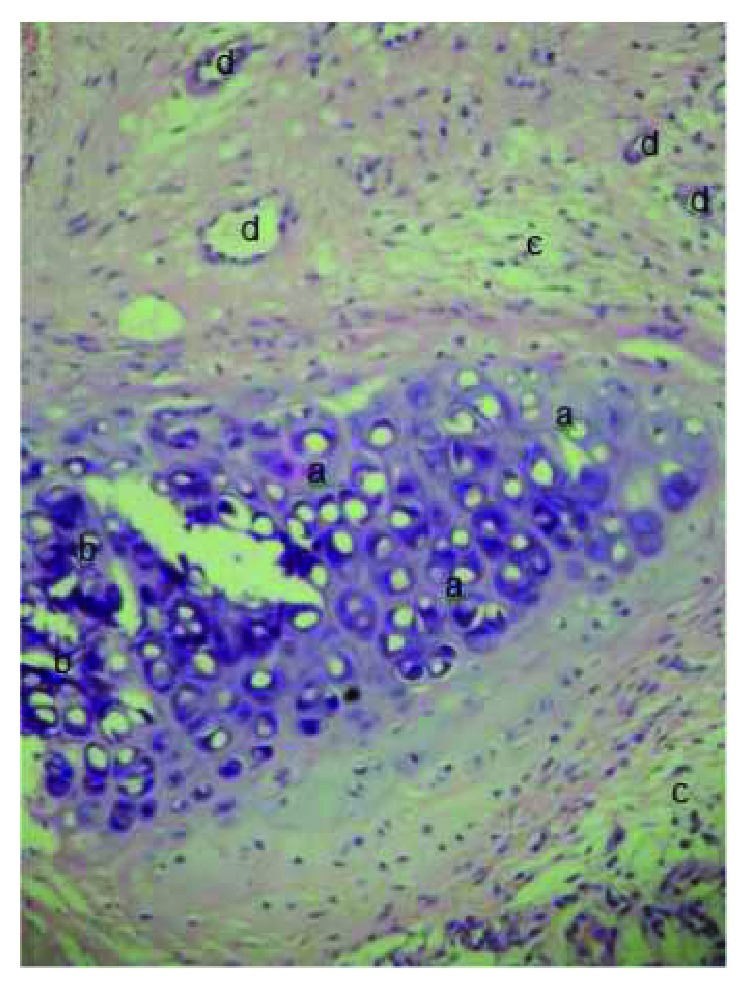
SIS group with immature cartilaginous tissue at the site of the tracheal defect, H&E (400x): (a) immature cartilaginous tissue (newly formed cartilage), (b) mature cartilaginous tissue, (c) connective tissue, and (d) blood vessels.

**Figure 16 fig16:**
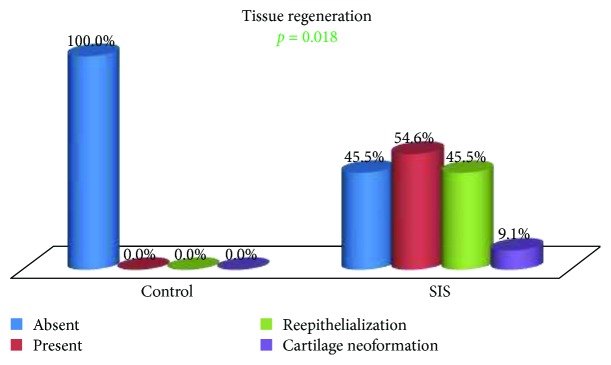
Tissue regeneration: group comparison.

**Figure 17:**
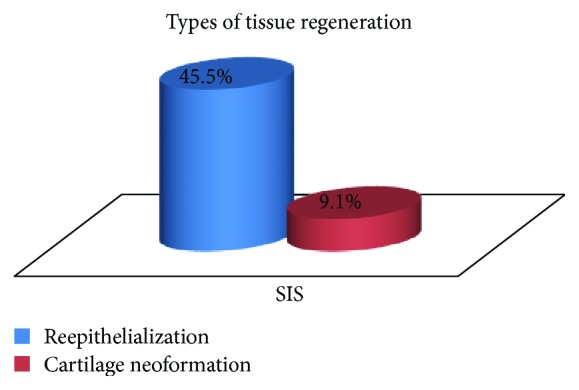
Tissue regeneration type within the SIS group.

A statistically significant difference in the presence of regenerated tissue was observed between the groups (*p* = 0.018) (Figure 12). In the control group, there was no detectable tissue regeneration in any of the samples, while in the SIS group, reepithelialization and newly formed cartilage were observed in 54.5% of the cases (Figures 13 [Fig fig14] [Fig fig15] [Fig fig16]–17).

## 4. Discussion

Although most tracheal lesions can be treated with resection and primary anastomosis, defects that do not allow such treatment remain a challenge in medical practice [8, 9, 17, 20, 25]. During the last few decades, efforts in tracheal reconstruction have been aimed at treating a variety of malignant and benign diseases [7, 26] such as tumors, trauma, infections, birth defects, and the most common injuries caused by tracheostomy and tracheal intubation [10, 14, 17]. Biological membranes are considered the best option to perform these reconstructions as they provide a scaffold for reepithelialization of the defect and proliferation of cartilage tissue through regeneration factors, which promote the cellular matrix growth of the host tissue [1, 17, 20, 24, 27].

The ideal material for replacing the tracheal wall must be airtight, hard, longitudinally flexible, coated with epithelial tissue, and highly vascularized to prevent infection and to allow healing [28]. There are many materials available for tissue replacement, for example, Dacron®, polyurethane, costal and ear cartilage flaps, and allograft aorta [2, 5, 17, 20]. However, they present many complications, such as infection, extrusion, obstruction, stenosis, and chronic graft rejection [12, 17, 20, 29]. Alternative possibilities, discussed by Grillo [30], are the use of synthetic structures such as stents, which have two distinct disadvantages: Correction of the lesion is permanently prevented, and severe complications may develop from the stent. Removable silicone stents also hinder curative treatment and may induce granulation, especially in the subglottic region. However, granulation is sometimes reversible in contrast to problems caused by permanent stents, which include stenosis of the trachea.

This study aimed at testing the use of SIS for repairing tracheal defects in rabbits by analyzing the dimensions of the tracheal defects after reconstruction and by evaluating the regeneration of the tracheal wall.

In order to perform this study, we created partial tracheal defects of 6 × 8 mm (48 mm^2^) dimensions in our rabbit model. The size of this defect was chosen because a 6 mm resection of a circumferential segment from a rabbit trachea would cause a reduction of approximately 30% of the normal rabbit tracheal transverse section area and a decrease in tracheal lumen of up to 40% may occur without compromising the respiratory dynamics [31]. As the aim of this study was not to evaluate respiratory dynamics, animals were not submitted to respiratory distress. We considered it appropriate to use an approximately 30% tracheal lumen reduction to avoid a stenosis progression.

A surprising result of this study was the shorter average perimeter and smaller average area of defects in the SIS group. This was not expected as there was interposition of a tissue graft between the edges of the tracheal defect, avoiding decrease in tracheal lumen area during reparation. The average perimeter of the tracheal defect in the SIS group decreased by 15.3% compared to that of the control group (*p* = 0.035), and the average area decreased by 21.8% (*p* = 0.151). A decrease in tracheal lumen of up to 40% may be acceptable without compromising respiratory dynamics [31], as seen in our experimental animals, all of which survived the surgical procedure with no signs of obstruction or stenosis of the airway. Although the decrease in average perimeter was small and, in the case of the average area, not statistically significant, it points to the inability of this method in maintaining the tracheal structure. We believe that the decrease might have occurred due to contraction at the healing stage secondary to the inflammation and fibrosis induction caused by surgical injury. It might be possible that the use of a temporary endotracheal support (stent) could prevent this stenosis.

Histological analysis revealed an inflammatory tissue in both groups with no statistically significant difference (*p* = 0.147), but the presence of inflammatory cells at the site of the graft in SIS group without any signs of necrosis confirms biocompatibility, lack of antigenicity, and absence of rejection and is in agreement with the observations of other in vivo studies with SIS [22, 27].

There was also no significant difference when comparing the presence of fibrosis (*p* = 1.000) and neovascularization (*p* = 0.603) between the two groups. However, it was important to verify the occurrence of neovascularization in almost 38% of the animals in the SIS group, a result that is in accordance with the findings of Poulose et al. [19], who previously described the occurrence of neovascularization in the SIS graft matrix used in the vena cava of pigs.

Probably the most important finding in this study is that related to tissue regeneration. There was a statistically significant difference (*p* = 0.018) in favor of the SIS group relating to reepithelialization and formation of new cartilage in more than one half of the cases (54.5%), while there was no tissue regeneration in any of the samples of the control group. The occurrence of this level of tissue interaction supports the utilization of SIS as a graft material to support tissue regeneration [12, 27].

The tissue engineering industry is still seeking the ideal tracheal substitute that provides cell-matrix interaction with receptor cells for the promotion of migration, proliferation, and reepithelization of defects [1].

Thus, porcine small intestinal submucosa, as a biodegradable tissue, is able to serve as a support for tissue remodeling [10, 15]. It has been widely used in many areas of medicine with satisfactory results in the regeneration of the aorta, vena cava, ligaments, skin, and other tissues [32]. The chemical and mechanical characteristics of SIS, combined with its low antigenicity, clearly make SIS a versatile and efficient option that appears to appropriately replace the tracheal tissue, although in our study it failed to prevent the occurrence of tracheal stenosis.

## 5. Conclusions

In summary, SIS showed some desirable properties when used as a graft material, partially replacing the tracheal wall in a rabbit model of tracheal injury, such as the capacity to promote tissue regeneration and to lower the risk of some serious postoperative complications like infection and graft extrusion or obstruction. However, it did not prevent the occurrence of tracheal stenosis but was successful in regenerating the tracheal wall by promoting efficient neovascularization, reepithelialization, and formation of new cartilage.

## Figures and Tables

**Table 1:** Tracheal defect perimeter: comparison between groups.

Group	*n*	Mean, mm	Median, mm	Minimum, mm	Maximum, mm	Standard deviation	*p* value
Control	8	1.703	1.697	1.342	2.005	0.229	
SIS	8	1.443	1.405	1.147	1.827	0.215	0.034

Student's *t*-test for independent samples, *p* < 0.05.

**Table 2:** Tracheal defect area: comparison between groups.

Group	*n*	Mean, mm^2^	Median, mm^2^	Minimum, mm^2^	Maximum, mm^2^	Standard deviation	*p* value
Control	8	0.151	0.151	0.090	0.197	0.039	
SIS	8	0.118	0.109	0.060	0.211	0.047	0.151

Student's *t*-test for independent samples, *p* < 0.05.

## Supplementary Materials

Supplementary Materials Annex 1: Committee of Ethics in Research in Animal decision.
