# Supplementary figures and images for: Regeneration of Tracheal Tissue in Partial Defects Using Porcine Small Intestinal Submucosa
Source: Stem Cells Int. 2018 Feb 26;2018:5102630. doi: 10.1155/2018/5102630 (PMC5846444; doi:10.1155/2018/5102630)

**ANNEX 1** - Committee of Ethics in Research in Animal decision.


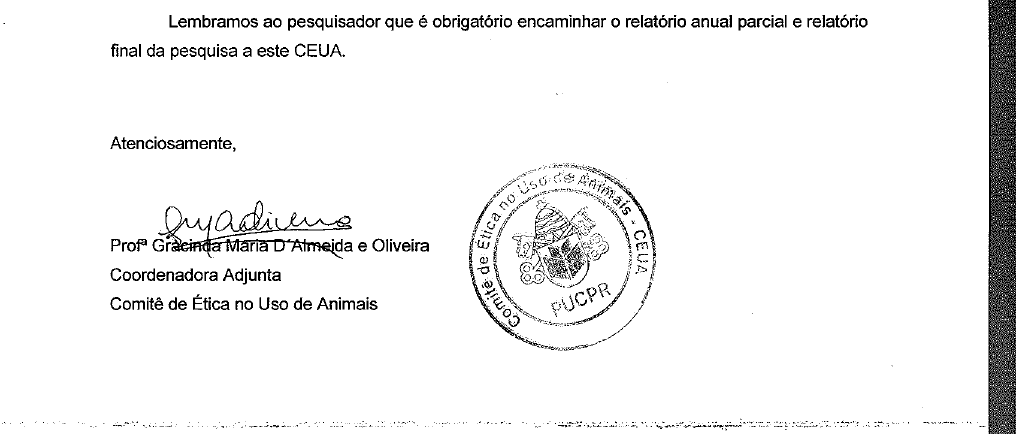

Supplement: Supplementary Materials — Annex 1: Committee of Ethics in Research in Animal decision. [file 5102630.f1.docx]
